# TET1 loss promotes malignant phenotypes in HeLa cells and is associated with altered PTEN–PI3K/AKT/mTOR signaling

**DOI:** 10.3389/fonc.2026.1963427

**Published:** 2026-09-24

**Authors:** Yan Liu, Lujia Liu, Lusheng Wang, Juan Ren, Fanle Meng, Mengling Chen, Xiaoyan Yao, Ping Zou, Jingjing Wu, Xiaoyan Zhou, Heng Tang, Weiwei Ma

**Affiliations:** 1 Obstetrics and Gynecology, Wanbei Coal Electric Group General Hospital, Suzhou, Anhui, China; 2 Central Laboratory, Wanbei Coal Electric Group General Hospital, Suzhou, Anhui, China; 3 Key Laboratory of Tumor Pathology of Suzhou, Suzhou, Anhui, China

**Keywords:** cell migration and invasion, cervical cancer, HeLa cells, PTEN–PI3K/AKT/mTOR signaling pathway, TET1

## Abstract

**Objective:**

To investigate the biological significance of ten–eleven translocation 1 (TET1) in cervical cancer and its association with PTEN–PI3K/AKT/mTOR signaling.

**Methods:**

Publicly available bulk and single-cell transcriptomic datasets were analyzed, with histology-restricted analyses focused on cervical squamous cell carcinoma (SCC). TET1 immunoreactivity was assessed in five paired cervical adenocarcinoma and adjacent non-neoplastic tissues using nuclear H-scores. TET1 loss of function was modeled in HeLa cells by shRNA-mediated knockdown, with Bobcat339 used as a complementary TET-family inhibitor. Cellular metabolic activity, clonogenicity, cell-cycle distribution, apoptosis, migration, and invasion were evaluated using CCK-8, colony-formation, flow-cytometric, wound-healing, and Transwell assays. Protein expression and phosphorylation were examined by western blotting.

**Results:**

SCC-restricted bulk analyses consistently showed lower TET1 expression in cervical SCC than in normal cervical tissues. In TCGA-CESC SCC, TET1 expression was not significantly associated with PTEN expression or the PI3K–AKT–mTOR transcriptional score after multiple-testing correction, whereas an inverse association with the apoptosis score remained significant. Patient-level pseudobulk analysis of 12 SCC cases likewise identified no pathway association that remained significant after false-discovery-rate correction. In five paired cervical adenocarcinoma cases, TET1 nuclear H-scores were lower in all tumor samples than in their matched adjacent tissues, although the difference did not reach statistical significance (exact P = 0.0625). In HeLa cells, TET1 depletion increased cellular metabolic activity and clonogenicity, altered cell-cycle distribution, reduced apoptosis, and enhanced migration and invasion. These changes were accompanied by reduced PTEN expression and increased phosphorylation of PI3K, AKT, and mTOR.

**Conclusion:**

Reduced TET1 expression was observed in SCC-restricted public cohorts, while TET1 depletion promoted malignant phenotypes in HeLa cells. The accompanying changes in PTEN expression and PI3K/AKT/mTOR phosphorylation identify a candidate signaling association that warrants validation through rescue experiments and locus-specific epigenetic studies.

## 1 Introduction

Cervical cancer remains a major gynecological malignancy worldwide and continues to contribute substantially to cancer-related morbidity and mortality among women, particularly in low- and middle-income countries (1, 2). Persistent infection with high-risk human papillomavirus (HPV) is the primary etiological factor in cervical carcinogenesis (3). However, only a small proportion of HPV-infected individuals ultimately develop malignant disease, indicating that HPV infection alone is insufficient for cervical carcinogenesis and that additional genetic and epigenetic alterations contribute to malignant transformation and progression (4). Although prevention, screening, and treatment have improved, patients with advanced or metastatic disease continue to have unfavorable outcomes, underscoring the need to better define the molecular drivers of cervical cancer progression.

Epigenetic dysregulation represents an important component of cervical carcinogenesis. Aberrant DNA methylation and hydroxymethylation can alter the transcriptional programs of tumor suppressor genes and oncogenes without changing the underlying DNA sequence (5, 6). Ten–eleven translocation 1 (TET1), a member of the TET family of dioxygenases, catalyzes the oxidation of 5-methylcytosine to 5-hydroxymethylcytosine and thereby participates in DNA demethylation (7, 8). The biological role of TET1 is highly context dependent across cancers, with both tumor-suppressive and oncogenic functions reported through the epigenetic regulation of genes involved in proliferation, apoptosis, epithelial–mesenchymal transition (EMT), and cell motility (9–11).

In cervical cancer, TET1 has been implicated in the regulation of cell migration and invasion through autophagy-related mechanisms (12). However, how TET1 loss relates to broader malignant phenotypes and cancer-associated signaling pathways remains incompletely understood. In particular, the signaling changes that accompany TET1 depletion and their relationship to cell growth, survival, migration, and invasion have not been fully characterized.

Dysregulation of the phosphatase and tensin homolog (PTEN)–phosphoinositide 3-kinase (PI3K)/AKT/mammalian target of rapamycin (mTOR) signaling axis has been implicated in cervical cancer cell growth, survival, and treatment response (13–15). PTEN negatively regulates this pathway, and reduced PTEN activity can facilitate downstream signaling. In gastric cancer, TET1 has been linked to PTEN promoter demethylation and re-expression (16), raising the possibility that a similar relationship may exist in cervical cancer. However, direct TET1-dependent epigenetic regulation of PTEN has not been established in this disease.

Therefore, we integrated publicly available bulk transcriptomic datasets, single-cell RNA-sequencing data, paired human cervical tissues, and a HeLa loss-of-function model to characterize TET1 dysregulation and examine its association with PTEN–PI3K/AKT/mTOR signaling and malignant cellular phenotypes. Histology-restricted analyses were applied where reliable subtype annotations were available. We further assessed the effects of TET1 depletion on cellular metabolic activity, clonogenicity, cell-cycle distribution, apoptosis, migration, and invasion.

## 2 Materials and methods

### 2.1 Public transcriptomic dataset analysis

Publicly available transcriptomic datasets were analyzed to assess TET1 expression and transcriptomic associations in cervical cancer. Histological annotations were reviewed before analysis. In GSE9750, 32 cervical SCC samples were retained, whereas one adenocarcinoma sample (GSM247876) was excluded; 24 normal cervical epithelial samples were used as controls. TET1 expression was represented by probe set 214862_x_at. SCC and normal tissues were compared using a two-sided Wilcoxon rank-sum test, with Cliff’s delta and a bootstrap 95% confidence interval reported as effect-size measures.

Uniformly processed TCGA-CESC and GTEx RNA-sequencing data were obtained from the UCSC Toil recompute dataset. The primary analysis included 252 TCGA cervical SCC samples after exclusion of 52 adenocarcinoma or adenosquamous tumors. Ten GTEx donor-level normal cervical tissue values were obtained by summarizing multiple samples from the same donor using the within-donor median. Expression was analyzed as log2(TPM + 1), and groups were compared using a two-sided Wilcoxon rank-sum test with Cliff’s delta and bootstrap 95% confidence intervals.

Within TCGA-CESC SCC tumors, the association between TET1 and PTEN expression was assessed using Spearman correlation with a bootstrap 95% confidence interval. Single-sample gene-set enrichment analysis (ssGSEA) scores were calculated for the prespecified Hallmark PI3K–AKT–mTOR, epithelial–mesenchymal transition (EMT), E2F targets, G2/M checkpoint, and apoptosis gene sets after excluding TET1 and PTEN. A cell-cycle composite was defined as the mean of the standardized E2F-target and G2/M-checkpoint scores. Spearman correlations between TET1 expression and the four pathway-level outcomes were calculated, with P values adjusted using the Benjamini–Hochberg method.

### 2.2 Single-cell RNA-sequencing analysis

Processed single-cell RNA-sequencing data were obtained from the Gene Expression Omnibus (GEO; GSE279998). To minimize histological heterogeneity, the analysis was restricted to 12 cervical squamous cell carcinoma samples. Following sample-wise quality control and doublet removal, 112,028 cells were retained for analysis. Gene expression was normalized and log-transformed, highly variable genes were selected, and dimensionality reduction was performed using principal-component analysis. Patient-associated batch effects were corrected using Harmony, followed by Leiden clustering and UMAP visualization. Major cell populations were annotated using canonical lineage markers and cluster-enriched genes.

Expression-derived copy-number variation (CNV) analysis was performed separately for each patient using infercnvpy, with non-epithelial immune and stromal cells from the same patient serving as reference populations. Epithelial cells with CNV scores exceeding the patient-specific 95th percentile of the reference distribution were classified as CNV-supported malignant epithelial cells, yielding 4,270 cells. TET1 transcript detectability was evaluated using original unique molecular identifier (UMI) counts; a raw TET1 UMI count > 0 was considered evidence of transcript detection and was not used for malignancy classification. Overall and patient-specific detection rates were calculated to characterize interpatient heterogeneity in TET1 detectability.

For patient-level pseudobulk analysis, raw UMI counts from CNV-supported malignant epithelial cells were summed within each of the 12 SCC patients, converted to counts per million, and transformed as log2(CPM + 1). ssGSEA scores were calculated using the Hallmark 2020 PI3K–AKT–mTOR, EMT, E2F-target, G2/M-checkpoint, and apoptosis gene sets (weighting exponent 0.25; normalization enabled), with TET1 and PTEN removed from the gene sets. The cell-cycle composite was defined as the mean of standardized E2F-target and G2/M-checkpoint scores. Patient-level TET1 expression was correlated with the four prespecified outcomes using Spearman correlation; 95% CIs were estimated from 10,000 bootstrap resamples, and P values were adjusted using the Benjamini–Hochberg method. Leave-one-patient-out analyses and exclusion of the patient contributing fewer than 100 malignant epithelial cells were performed as sensitivity analyses. Given the limited sample size (n = 12), these analyses were considered exploratory.

### 2.3 Immunohistochemistry (IHC)

Five evaluable pairs of cervical adenocarcinoma and adjacent non-neoplastic cervical tissue were obtained from five female patients who underwent surgery at Wanbei Coal Electric Group General Hospital between June 2021 and April 2023. Cases were included if paired formalin-fixed, paraffin-embedded tissues were available for evaluation. The patients were 34–60 years old, and all five tumors were pathologically confirmed cervical adenocarcinomas. Two cases were accompanied by focal high-grade squamous intraepithelial lesion (HSIL/CIN2), and one was documented as usual-type cervical adenocarcinoma. Individual clinicopathological diagnoses and paired H-scores are provided in **Supplementary Table S1**. None of the patients received chemotherapy or radiotherapy before surgery. The study was approved by the Ethics Committee of Wanbei Coal Electric Group General Hospital, and written informed consent was obtained in accordance with the Declaration of Helsinki.

Tissue sections were deparaffinized and rehydrated before immunohistochemical staining. Antigen retrieval was performed in citrate buffer (pH 6.0) using a microwave oven. Sections were heated at high power for 5 min, briefly cooled, heated at medium-high power for another 5 min, and then cooled to room temperature. Endogenous peroxidase activity was blocked with 3% hydrogen peroxide for 10 min, followed by blocking with normal goat serum. Sections were incubated overnight at 4 °Cwith an anti-TET1 antibody (Abcam, catalog no. ab272900; 1:500). After washing, sections were incubated with an HRP-conjugated secondary antibody, visualized with 3,3′-diaminobenzidine, and counterstained with hematoxylin. Representative paired fields were acquired using a Leica DM2000 microscope (Leica Microsystems CMS GmbH).

For quantitative assessment, digital images were analyzed by H-DAB color deconvolution and automated nuclear segmentation using fixed thresholds applied consistently across paired tissues. Nuclei were classified as negative, weak, moderate, or strong, and a nuclear H-score was calculated as 1 × percentage weak + 2 × percentage moderate + 3 × percentage strong (range, 0–300). One H-score was derived for each tissue, and the five paired values were compared using an exact two-sided Wilcoxon signed-rank test.

### 2.4 Cell culture and stable knockdown of TET1

HeLa cells were cultured in Dulbecco’s modified Eagle’s medium (DMEM; Gibco, USA) supplemented with 10% fetal bovine serum and 1% penicillin–streptomycin at 37 °C in 5% CO_2_. 5 (5′-AGCAACATGATTGTGATTATA-3′), sh-TET1-1490 (5′-CGTCTCCTACCTCTGTAATAA-3′), sh-TET1-2430 (5′-TGTGCCTCTGGAGGTTATAAA-3′), or a non-targeting control (5′-GTTCTCCGAACGTGTCACGT-3′) were purchased from Shanghai GenePharma Co., Ltd. (Shanghai, China) and introduced into HeLa cells by lentiviral transduction at a multiplicity of infection (MOI) of 4 using viral stocks with a titer of 3 × 10^8^ TU/mL. Stable knockdown cells were selected, and knockdown efficiency was evaluated by qRT-PCR and western blotting. For complementary pharmacological experiments, cells were treated with 15 μM Bobcat339 (MedChemExpress, USA), a TET-family inhibitor used as a pharmacological comparator rather than as TET1-specific validation.

### 2.5 CCK-8 cellular metabolic activity assay

Cellular metabolic activity was assessed using a Cell Counting Kit-8 (CCK-8; Solarbio, China). sh-NC cells, sh-TET1 cells, and sh-NC cells treated with 15 μM Bobcat339 were seeded in 96-well plates at 5 × 10³ cells per well. Separate wells were assigned to the 24-, 48-, and 72-h measurements to avoid repeated measurements of the same wells. At each designated time point, 10 μL CCK-8 reagent was added to each well, followed by incubation for 2 h at 37°C. Technical replicate wells were averaged within each independent experiment, and the independent experiment was used as the statistical unit. Values were normalized to the sh-NC group at 24 h.

### 2.6 Colony formation assay

For colony formation, sh-NC and sh-TET1 cells were seeded in 6-well plates at 500 cells per well, and 15 μM Bobcat339 was added where indicated. Cells were cultured for exactly 14 days, with medium replaced every 3 days. Because the assay was designed as a single endpoint experiment, no intermediate images were acquired. Colonies were fixed with 4% paraformaldehyde, stained with 0.1% crystal violet, and those containing more than 50 cells were counted under a light microscope.

### 2.7 Cell cycle analysis

Cell cycle distribution was analyzed by flow cytometry. Briefly, cells from different groups were harvested, washed with PBS, and fixed in 70% ethanol at 4 °C overnight. After fixation, cells were treated with RNase A (100 μg/mL) and stained with propidium iodide (PI, 50 μg/mL) for 30 min in the dark. The proportions of cells in the G0/G1, S, and G2/M phases were determined using a Beckman Coulter flow cytometer and ModFit software.

### 2.8 Apoptosis assay

Cell apoptosis was evaluated using an Annexin V-FITC/propidium iodide (PI) apoptosis detection kit (Beyotime Biotechnology, China). Cells were harvested, washed twice with cold PBS, and resuspended in binding buffer. Annexin V-FITC and PI were added according to the manufacturer’s instructions, and samples were analyzed by flow cytometry (**Figure 1A**). Early- and late-apoptotic populations were combined and expressed as a percentage of total cells.

**Figure 1:**
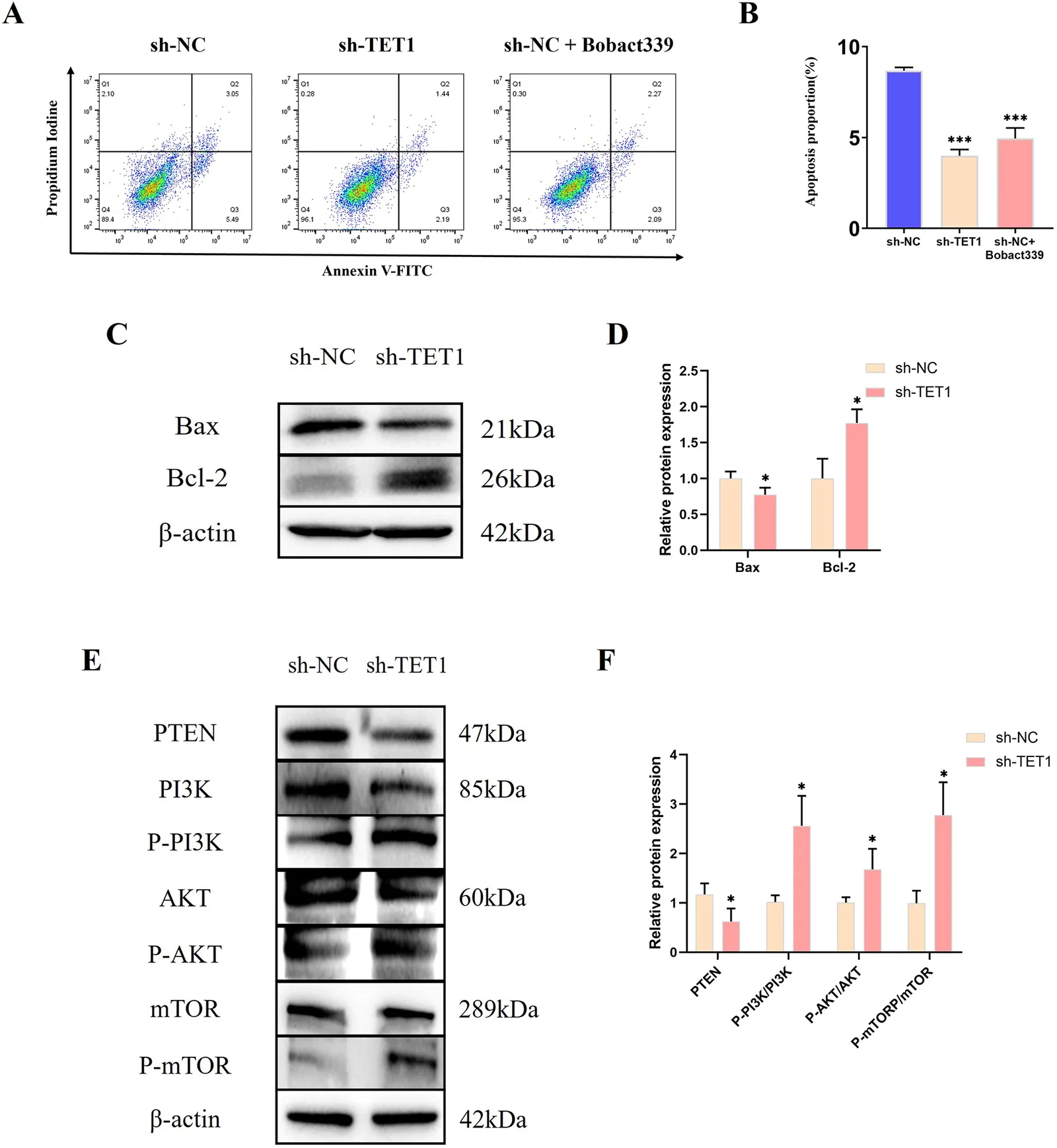
TET1 depletion is associated with reduced apoptosis and altered PTEN–PI3K/AKT/mTOR signaling in HeLa cells. **(A)** Representative Annexin V-FITC/PI flow-cytometric apoptosis plots for sh-NC, sh-TET1, and sh-NC + Bobcat339 groups. **(B)** Combined early- and late-apoptotic fractions. **(C)** Bax and Bcl-2 western blots and **(D)** densitometry after TET1 knockdown. **(E)** PTEN and total and phosphorylated PI3K, AKT, and mTOR western blots and **(F)** densitometry. Data are mean ± SD from three independent experiments. Panel **(B)** was analyzed by one-way ANOVA with Tukey adjustment; panels **(D)** and **(F)** were analyzed by two-sided Student’s t tests. Bobcat339 was included only in the phenotypic apoptosis analysis in panels **(A)** and **(B)**; panels **(C–F)** evaluated the genetic knockdown comparison without Bobcat339. *P < 0.05; **P < 0.01; ***P < 0.001.

### 2.9 Wound healing assay

Cell migration was assessed using a wound healing assay. Cells were seeded into 6-well plates and cultured to near confluence. A linear wound was generated using a sterile 200 μL pipette tip. After washing with PBS, serum-free medium containing or lacking 15 μM Bobcat339 was added. Images were captured at 0 h and 24 h using an inverted microscope (Leica Microsystems CMS GmbH). Wound closure areas were quantified using ImageJ software.

### 2.10 Transwell migration and invasion assays

Cell migration and invasion assays were performed using Transwell chambers with 8 μm pore-size membranes (Corning, USA). For invasion assays, the upper chambers were precoated with Matrigel (Corning, USA). Cells (5 × 10^4^ cells/well) were seeded in the upper chamber in serum-free medium, whereas medium containing 10% FBS was added to the lower chambers. Bobcat339 (15 μM) was added where indicated. After 24 h of incubation, non-migrated or non-invaded cells on the upper surface were removed using cotton swabs. Cells on the lower surface were fixed with paraformaldehyde, stained with crystal violet, and counted in five randomly selected microscopic fields.

### 2.11 Quantitative real-time PCR (qRT-PCR)

Total RNA was extracted from HeLa cells using TRIzol reagent according to the manufacturer’s instructions. RNA concentration and purity were measured using a NanoDrop spectrophotometer (Thermo Fisher Scientific, USA). One microgram of total RNA was reverse-transcribed into cDNA using PrimeScript RT Master Mix (Takara, Japan). qRT-PCR was performed using SYBR Green Master Mix (Biosharp, China) on a real-time PCR system. The thermal cycling conditions were 95 °C for 5 min, followed by 40 cycles of 95 °C for 10 s and 60 °C for 30 s. Relative gene expression levels were normalized to β-actin using the 2^−ΔΔCt method. All reactions were performed in triplicate.

*The primer sequences used in this study were as follows:*

*TET1 forward: 5′-ATCCTTGCTGTGTAGAGTTC-3′;*
*TET1 reverse: 5′-AGTATTGGTGATATGCTTCCTAA-3′;*
*β-actin forward: 5′-CATTCCAAATATGAGATGCGTT-3′;*
*β-actin reverse: 5′-TACACGAAAGCAATGCTATCAC-3′.*

### 2.12 Western blot analysis

Total protein was extracted using RIPA lysis buffer supplemented with protease and phosphatase inhibitors. Protein concentrations were determined using a BCA protein assay kit. Equal amounts of protein were separated by SDS-PAGE and transferred onto PVDF membranes. Membranes were blocked with 5% non-fat milk for 1 h at room temperature and incubated overnight at 4 °C with primary antibodies against TET1 (#AB272900, Abcam), CDK4 (#AB108357, Abcam), Cyclin D1 (#AB134175, Abcam), Bax (#60267, Proteintech), Bcl-2 (#26593, Proteintech), PTEN (#60300, Proteintech), PI3K (#60225, Proteintech), phospho-PI3 kinase p85 (Tyr458)/p55 (Tyr199) (4228S, Cell Signaling Technology; 1:1000), AKT (#60203, Proteintech), phospho-AKT (Ser473) (66444, Proteintech; 1:2000), mTOR (#66888, Proteintech), phospho-mTOR (Ser2448) (67778, Proteintech; 1:2000), MMP2 (#AF5330, Affinity Biosciences), MMP9 (#AF5228, Affinity Biosciences), E-cadherin (#3195S, Cell Signaling Technology), N-cadherin (#13116S, Cell Signaling Technology), Snail (#A5243, ABclonal) and Vimentin (#5741S, Cell Signaling Technology).

After washing with TBST, membranes were incubated with HRP-conjugated anti-rabbit secondary antibody (#RGAR001, Proteintech) or anti-mouse secondary antibody (#RGAM001, Proteintech) for 1 h at room temperature. Protein bands were visualized using enhanced chemiluminescence (ECL) reagents, with β-actin serving as the loading control.

### 2.13 Statistical analysis

All *in vitro* experiments were independently repeated at least three times, and data are presented as mean ± SD. Technical replicates were averaged within each independent experiment before statistical analysis. Two-group experimental comparisons were analyzed using two-sided Student’s t tests, whereas three-group endpoint experiments were analyzed using one-way ANOVA followed by Tukey’s multiple-comparisons test. Because separate wells were used at 24, 48, and 72 h in the CCK-8 assay, cellular metabolic activity was analyzed using an ordinary two-way ANOVA including group, time, and group × time effects, followed by Dunnett-adjusted comparisons with sh-NC at each time point. Cell-cycle fractions were analyzed separately for each phase using one-way ANOVA with Tukey adjustment. Paired IHC H-scores were analyzed using an exact two-sided Wilcoxon signed-rank test. Public-cohort group differences were evaluated using two-sided Wilcoxon rank-sum tests with Cliff’s delta, whereas Spearman correlations were reported with bootstrap 95% confidence intervals. Benjamini–Hochberg false-discovery-rate correction was applied across prespecified pathway tests. GraphPad Prism 8.0 and R were used for statistical analyses as appropriate. Two-sided *P* < 0.05 or FDR < 0.05 was considered statistically significant.

## 3 Results

### 3.1 SCC-restricted public-cohort analysis of TET1 expression and transcriptomic associations

TET1 expression was first examined in histology-restricted public cohorts. In GSE9750, SCC samples had lower TET1 expression than normal cervical epithelium (SCC n = 32, median = 3.355; normal n = 24, median = 4.353; Wilcoxon P = 0.020; Cliff’s δ = −0.367, 95% CI −0.641 to −0.077; **Figure 2A**). In the TCGA-CESC/GTEx comparison, TCGA cervical SCC samples also had lower TET1 expression than donor-level GTEx normal cervical tissues (SCC n = 252, median = 0.124; normal n = 10, median = 0.475; P = 0.00178; Cliff’s δ = −0.583, 95% CI −0.805 to −0.318; **Figure 2B**).

**Figure 2:**
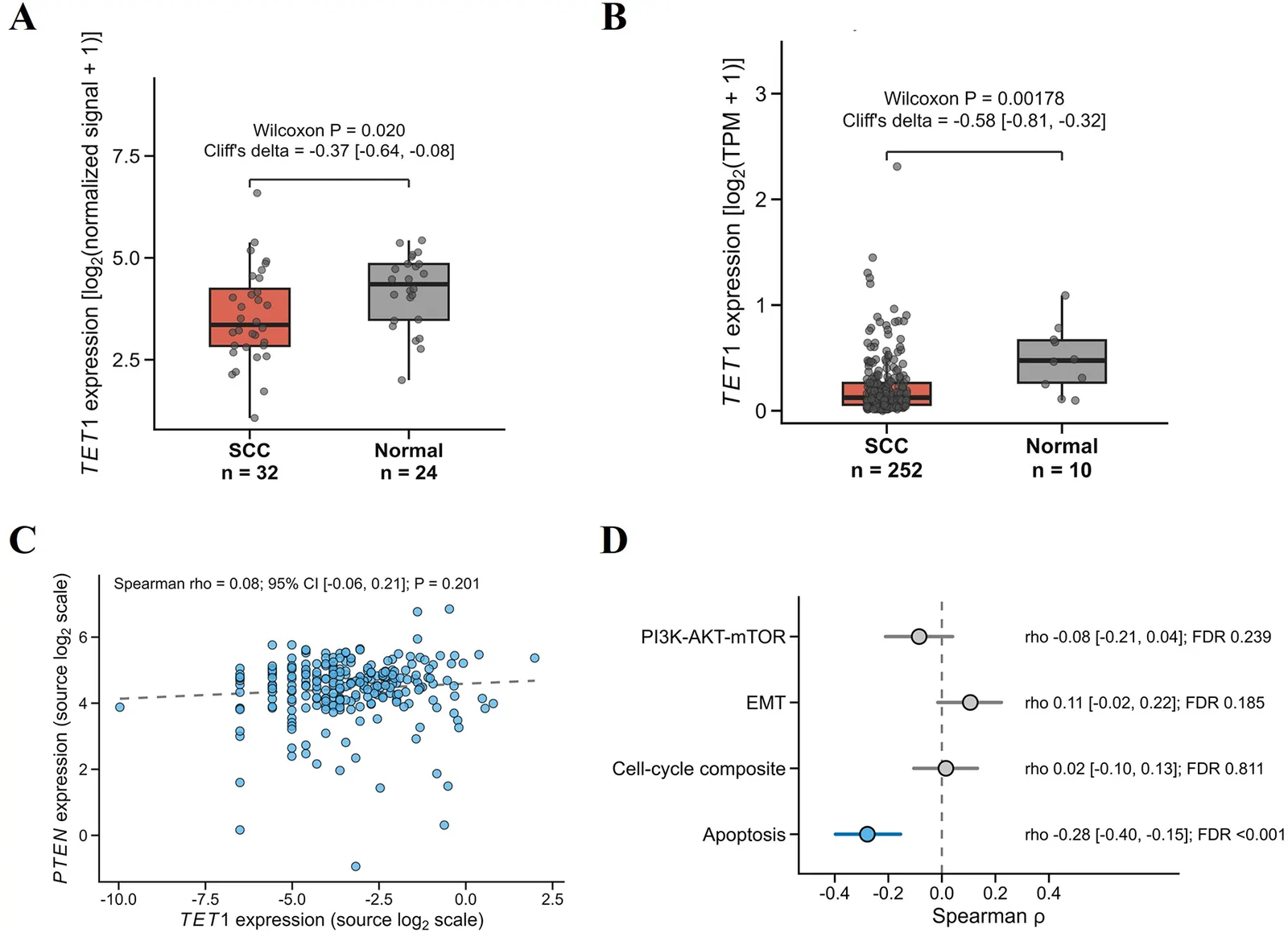
SCC-restricted public-cohort analyses of TET1 expression and transcriptomic associations. **(A)** TET1 expression in cervical SCC (n = 32) and normal cervical epithelium (n = 24) in GSE9750 (two-sided Wilcoxon rank-sum P = 0.020; Cliff’s δ = −0.367, 95% CI −0.641 to −0.077). **(B)** TET1 expression in TCGA-CESC primary SCC (n = 252) and GTEx donor-level normal cervical tissues (n = 10; P = 0.00178; Cliff’s δ = −0.583, 95% CI −0.805 to −0.318). **(C)** Spearman association between TET1 and PTEN in TCGA-CESC SCC (ρ = 0.081, 95% CI −0.055 to 0.211; P = 0.201). **(D)** Spearman associations between TET1 and PI3K–AKT–mTOR, EMT, cell-cycle composite, and apoptosis scores. Only the inverse association with apoptosis remained significant after Benjamini–Hochberg correction (ρ = −0.278, 95% CI −0.398 to −0.151; FDR < 0.001). Points and horizontal lines indicate ρ and bootstrap 95% CIs; the dashed line indicates ρ = 0.

Within TCGA-CESC SCC tumors, TET1 expression was not significantly correlated with PTEN expression (Spearman ρ = 0.081, 95% CI −0.055 to 0.211; P = 0.201; **Figure 2C**). Associations with PI3K–AKT–mTOR (ρ = −0.085, FDR = 0.239), EMT (ρ = 0.106, FDR = 0.185), and the cell-cycle composite (ρ = 0.015, FDR = 0.811) were not significant after correction. An inverse association with the apoptosis score remained significant (ρ = −0.278, 95% CI −0.398 to −0.151; FDR < 0.001; **Figure 2D**). Together, these analyses support reduced TET1 expression in cervical SCC but not a significant transcriptomic association between TET1 and PTEN–PI3K/AKT/mTOR signaling.

### 3.2 Single-cell localization and interpatient heterogeneity of TET1 detectability in cervical squamous cell carcinoma

To characterize the cellular distribution of TET1 in cervical squamous cell carcinoma (SCC), we analyzed 112,028 quality-controlled cells from 12 patients in GSE279998. UMAP visualization and canonical lineage markers identified 11 major epithelial, immune, and stromal cell populations (**Figure 3A**). Expression-derived copy-number variation analysis further identified 4,270 CNV-supported malignant epithelial cells, together with 23,068 epithelial cells without CNV support and 84,690 non-epithelial cells (**Figure 3B**).

**Figure 3:**
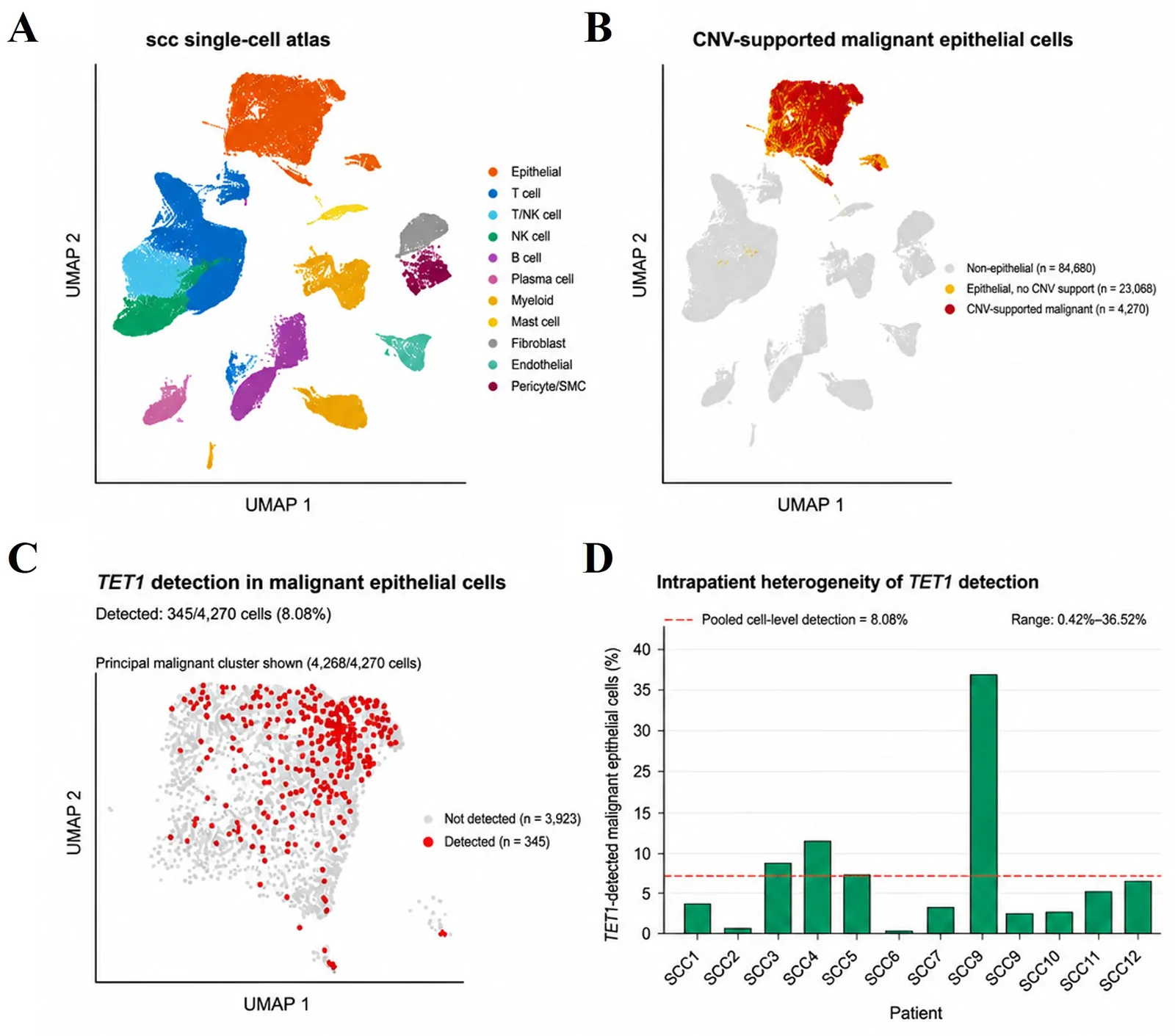
Single-cell localization and interpatient heterogeneity of TET1 detectability in cervical squamous cell carcinoma. **(A)** UMAP visualization of 112,028 quality-controlled cells from 12 patients with cervical squamous cell carcinoma in GSE279998, annotated into 11 major cell populations. **(B)** Identification of 4,270 CNV-supported malignant epithelial cells based on expression-derived copy-number variation profiles. **(C)** TET1 transcript detection in CNV-supported malignant epithelial cells. TET1 was detected in 345 of 4,270 cells (8.08%), based on raw UMI counts > 0. **(D)** Patient-level TET1 detection rates across the 12 samples, ranging from 0.42% to 36.52%. The dashed line indicates the pooled detection rate of 8.08%.

Within the CNV-supported malignant epithelial population, TET1 transcripts were detected in 345 of 4,270 cells (8.08%), using a raw UMI count > 0 as the detection criterion (**Figure 3C**). Patient-specific detection rates ranged from 0.42% to 36.52% across the 12 patients (**Figure 3D**), indicating substantial interpatient heterogeneity.

At the patient level, none of the four pseudobulk associations survived false-discovery-rate correction: PI3K–AKT–mTOR (ρ = −0.028, P = 0.931, FDR = 0.931), EMT (ρ = 0.552, P = 0.063, FDR = 0.250), cell-cycle composite (ρ = 0.084, P = 0.795, FDR = 0.931), and apoptosis (ρ = 0.294, P = 0.354, FDR = 0.708). The EMT association remained positive but non-significant after excluding the patient with fewer than 100 malignant epithelial cells (ρ = 0.536, P = 0.089, FDR = 0.356), and leave-one-patient-out estimates remained positive (ρ range, 0.455–0.791).

### 3.3 Exploratory paired tissue assessment and TET1 silencing in HeLa cells

TET1 immunoreactivity was evaluated in five paired cervical adenocarcinoma and adjacent non-neoplastic tissues (**Supplementary Table S1**). Nuclear H-scores were lower in the tumor sample of each pair. The median H-score was 29.2 in tumor tissue and 124.7 in adjacent tissue, and the median paired difference (adjacent minus tumor) was 77.8. The paired difference did not reach statistical significance in this small exploratory series (exact two-sided Wilcoxon signed-rank P = 0.0625; **Figures 4A, B**).

**Figure 4:**
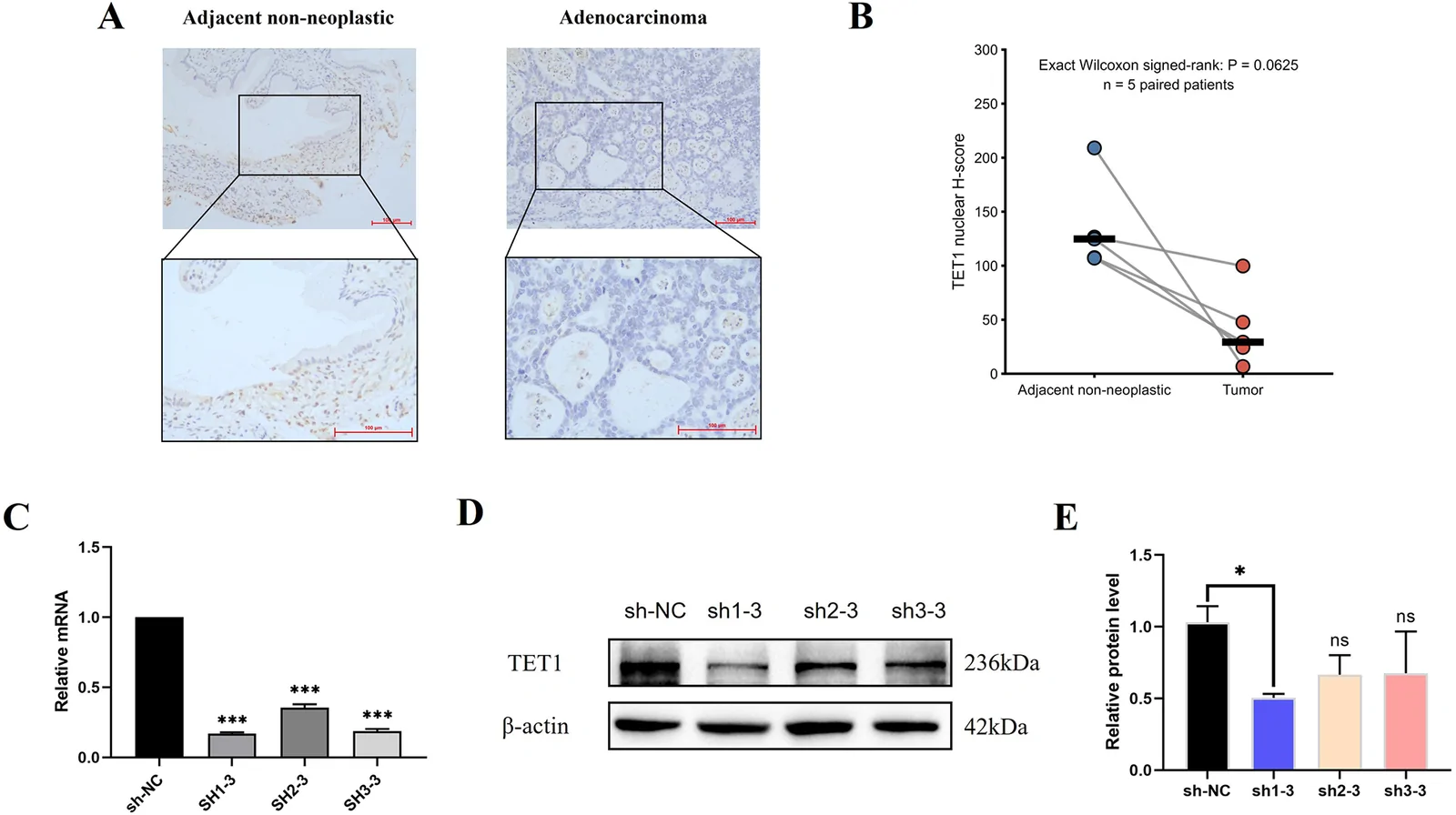
Exploratory paired TET1 immunohistochemistry in cervical adenocarcinoma and shRNA-mediated silencing in HeLa cells. **(A)** Representative TET1 staining in cervical adenocarcinoma and paired adjacent non-neoplastic tissues; boxed regions are shown at higher magnification and scale bars are indicated. **(B)** Image-derived nuclear TET1 H-scores in five paired cases. Lines connect tissues from the same patient and horizontal bars indicate medians (exact two-sided Wilcoxon signed-rank P = 0.0625). **(C)** TET1 mRNA after sh-NC or three TET1-targeting shRNAs (sh1-3, sh2-3, and sh3-3). **(D)** Representative TET1 western blot and **(E)** densitometric quantification relative to β-actin. Data in **(C)** and **(E)** are mean ± SD from three independent experiments and were analyzed by one-way ANOVA with Tukey adjustment. ns, not significant; *P < 0.05; ***P < 0.001 versus sh-NC.

To establish a TET1 loss-of-function model, three independent shRNAs targeting TET1 were introduced into HeLa cells. qRT-PCR showed reduced TET1 mRNA expression with all three constructs (**Figure 4C**). Western blotting showed the greatest protein reduction with sh1-3, whereas sh2–3 and sh3–3 had comparatively limited protein-level effects (**Figures 4D, E**). Accordingly, sh1–3 was selected for subsequent loss-of-function experiments.

### 3.4 TET1 suppression increases cellular metabolic activity and alters cell-cycle distribution in HeLa cells

The CCK-8 time course, performed in separate wells at each time point, showed higher cellular metabolic activity in sh-TET1 cells at later time points than in sh-NC cells (**Figure 5A**). Bobcat339 treatment produced a similar pattern (**Figure 5A**). Colony-formation assays performed at the 14-day endpoint showed increased colony numbers after TET1 knockdown and after Bobcat339 treatment (**Figures 5B, C**), indicating greater clonogenic capacity.

**Figure 5:**
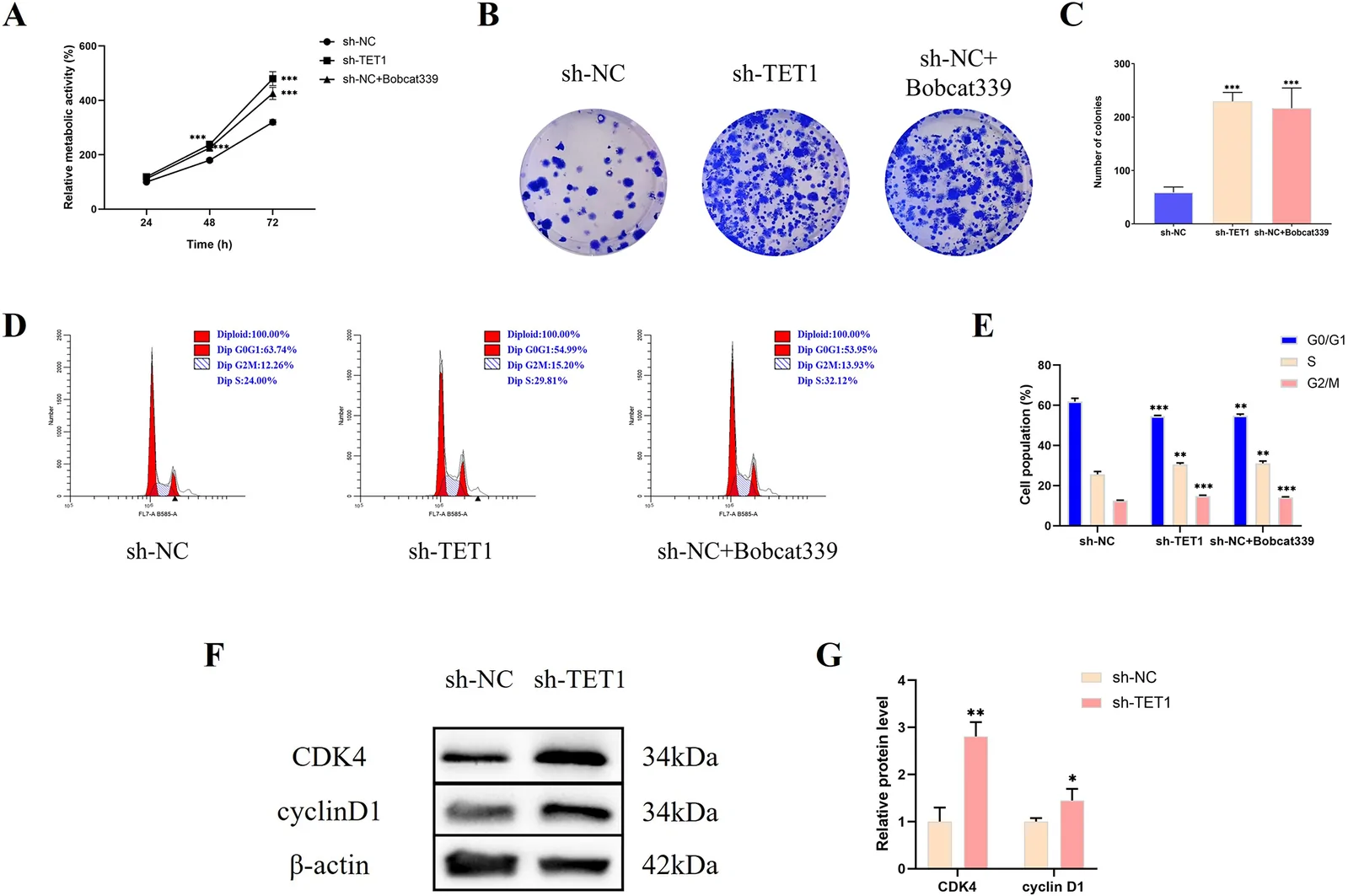
TET1 suppression increases cellular metabolic activity and alters cell-cycle distribution in HeLa cells. **(A)** Relative CCK-8 metabolic activity in sh-NC, sh-TET1, and sh-NC + Bobcat339 groups at 24, 48, and 72 h. Separate wells were used at each time point; technical replicate wells were averaged within each of three independent experiments. Values were normalized to sh-NC at 24 h. **(B)** Representative 14-day colony-formation endpoints and **(C)** colony counts. **(D)** Representative cell-cycle profiles and **(E)** G0/G1-, S-, and G2/M-phase fractions. Corresponding numerical values are provided in **Supplementary Table S2**. **(F)** CDK4 and Cyclin D1 western blots and **(G)** densitometry relative to β-actin. Data are mean ± SD. Panel **(A)** was analyzed by ordinary two-way ANOVA containing group, time, and group × time terms, followed by Dunnett-adjusted comparisons with sh-NC at each time point; **(C)** and phase-specific data in **(E)** by one-way ANOVA with Tukey adjustment; and **(G)** by two-sided Student’s t tests. Bobcat339 was used as a complementary TET-family inhibitor. ns, not significant; *P < 0.05; **P < 0.01; ***P < 0.001 versus the corresponding sh-NC group.

Flow-cytometric analysis showed altered cell-cycle distributions after TET1 knockdown and Bobcat339 treatment (**Figures 5D, E**). Phase-specific mean ± SD values from three independent experiments are provided in **Supplementary Table S2**. Western blotting further showed increased CDK4 and Cyclin D1 expression following TET1 knockdown (**Figures 5F, G**).

### 3.5 TET1 depletion is associated with reduced apoptosis and altered PTEN–PI3K/AKT/mTOR signaling in HeLa cells

Annexin V-FITC/PI flow cytometry showed a lower combined early- and late-apoptotic fraction in sh-TET1 cells than in sh-NC cells (**Figures 1A, B**). Bobcat339 treatment produced a similar reduction in the apoptotic fraction. In the genetic knockdown comparison, Bax expression decreased and Bcl-2 expression increased (**Figures 1C, D**), consistent with reduced apoptotic signaling following TET1 depletion.

Western blotting showed reduced PTEN protein expression following TET1 knockdown, accompanied by increased phosphorylation of PI3K, AKT, and mTOR, whereas the corresponding total protein levels showed no marked changes (**Figures 1E, F**).

### 3.6 TET1 loss enhances HeLa cell migration and invasion

Wound-healing and Transwell assays were used to examine the migratory and invasive phenotypes associated with TET1 suppression. sh-TET1 cells showed increased wound closure and greater migration and invasion than sh-NC cells (**Figures 6A–D**). Bobcat339 treatment produced concordant increases in migration and invasion.

**Figure 6:**
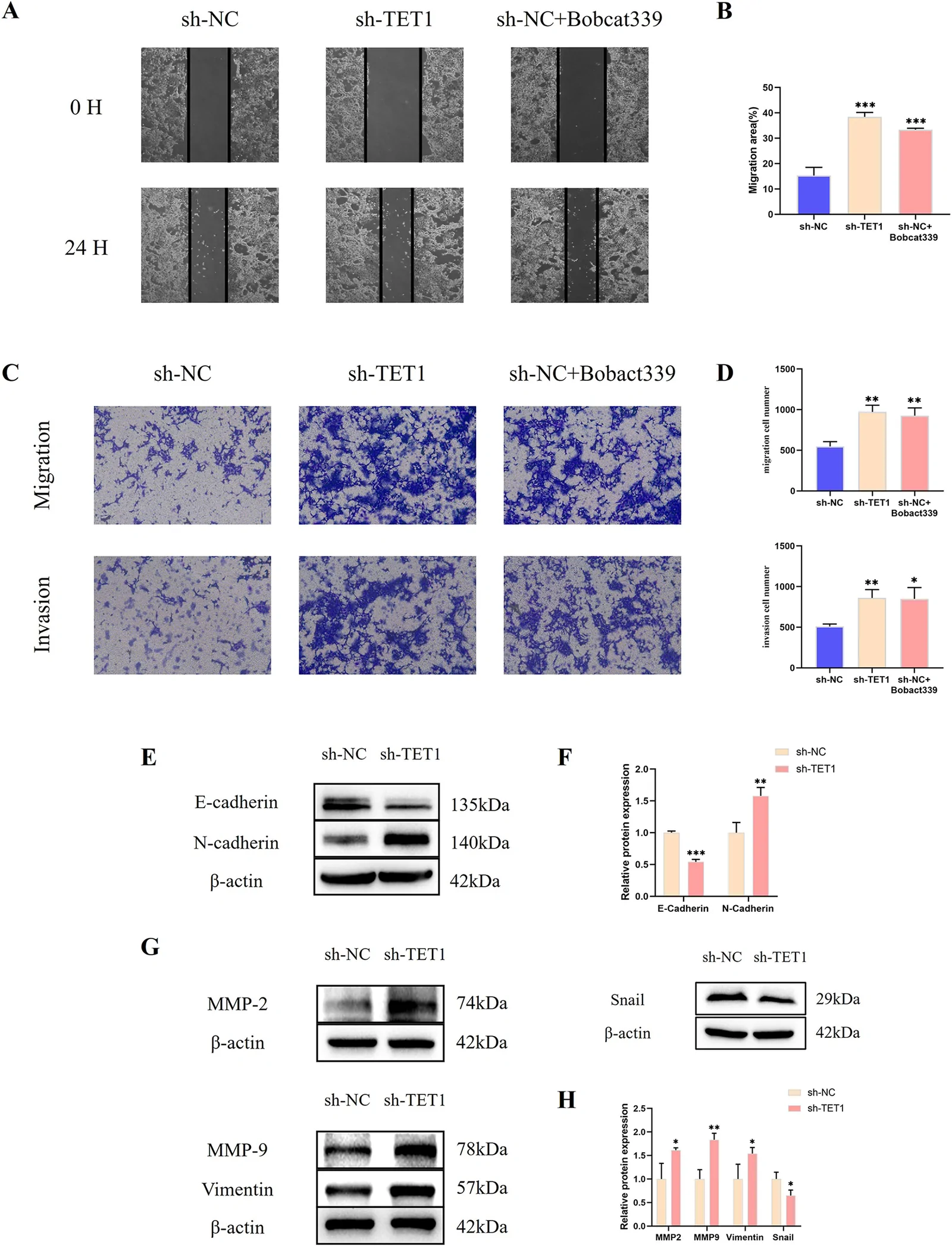
TET1 suppression enhances HeLa cell migration and invasion. **(A, B)** Wound healing; **(C, D)** Transwell migration and invasion; **(E, F)** E-cadherin and N-cadherin; and **(G, H)** MMP-2, MMP-9, Vimentin, and Snail. Images and corresponding quantification are shown. Data are mean ± SD from three independent experiments. Panels **(B)** and **(D)** used one-way ANOVA with Tukey adjustment; panels **(F)** and **(H)** used two-sided Student’s t tests. Bobcat339 was included only in panels **(A–D)**; panels **(E–H)** compared sh-NC and sh-TET1. *P < 0.05; **P < 0.01; ***P < 0.001.

To characterize the molecular changes associated with these phenotypes, migration- and invasion-related proteins were examined by western blotting. TET1 depletion decreased E-cadherin expression and increased N-cadherin expression (**Figures 6E, F**). In addition, MMP-2, MMP-9, Vimentin, and Snail expression levels were increased following TET1 knockdown (**Figures 6G, H**). These protein changes were consistent with the enhanced migratory and invasive phenotypes observed after TET1 depletion.

**Figure 7** summarizes the observed associations between TET1 depletion, altered PTEN–PI3K/AKT/mTOR signaling, and malignant phenotypes in HeLa cells.

**Figure 7:**
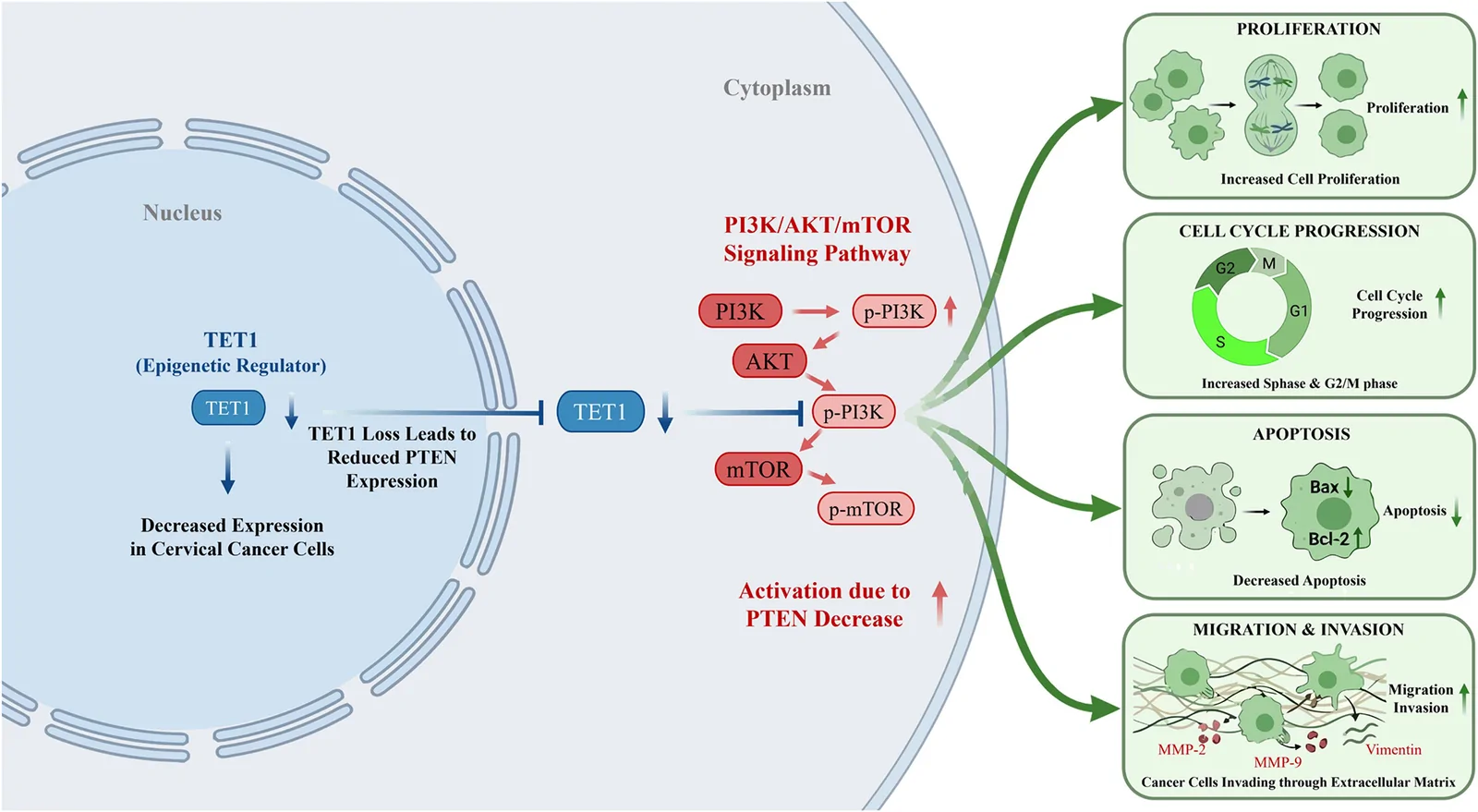
Proposed model illustrating the association between TET1 loss, PTEN–PI3K/AKT/mTOR signaling alterations, and malignant phenotypes in HeLa cells.

## 4 Discussion

This study integrated SCC-restricted public-cohort analyses, SCC single-cell data, paired cervical adenocarcinoma tissues, and loss-of-function experiments in adenocarcinoma-derived HeLa cells. TET1 expression was reduced in two independent SCC-restricted public comparisons, while all five adenocarcinoma tissue pairs showed lower nuclear TET1 H-scores in tumor than in adjacent non-neoplastic tissue, although the paired difference was not statistically significant. In HeLa cells, TET1 depletion increased cellular metabolic activity and clonogenicity, altered cell-cycle distribution, reduced apoptosis, and enhanced migration and invasion, accompanied by lower PTEN expression and increased phosphorylation of PI3K, AKT, and mTOR.

The biological role of TET1 is context dependent across cancers, with tumor-suppressive and tumor-promoting effects reported in different settings (9–11). In cervical cancer, previous work linked TET1 depletion to proliferation, migration, invasion, EMT-associated changes, xenograft growth, and autophagy-related epigenetic regulation (12). The present study extends these observations by combining histology-restricted cohort analyses, patient-level single-cell assessment, quantitative paired-tissue evaluation, apoptosis measurements, and PTEN–PI3K/AKT/mTOR protein analysis.

The SCC-restricted GSE9750 and TCGA-CESC/GTEx analyses consistently supported lower TET1 expression in cervical SCC. However, within TCGA-CESC SCC, TET1 expression was not significantly associated with PTEN expression or the PI3K–AKT–mTOR transcriptional score after multiple-testing correction; among the four prespecified pathway outcomes, only the inverse association with the apoptosis score remained significant. Single-cell analysis further showed that TET1 transcripts were detectable in only a subset of CNV-supported malignant epithelial cells and varied markedly among patients. Consistent with this heterogeneity, none of the patient-level pseudobulk associations with PI3K–AKT–mTOR, EMT, cell cycle, or apoptosis remained significant after false-discovery-rate correction. Together, these findings support cohort-level TET1 dysregulation but do not provide independent patient-level evidence for a PTEN–PI3K/AKT/mTOR transcriptional relationship.

The cell-model findings nevertheless identified a potentially relevant molecular association between TET1 depletion and PTEN–PI3K/AKT/mTOR signaling. PTEN is a negative regulator of this pathway, which contributes to tumor-cell growth, survival, metabolism, and motility (17). TET1 knockdown was accompanied by lower PTEN expression and increased phosphorylation of PI3K, AKT, and mTOR, together with reduced apoptosis and enhanced migration and invasion. These functional changes were also accompanied by decreased E-cadherin and increased N-cadherin, Vimentin, Snail, MMP-2, and MMP-9 expression, consistent with a more invasive phenotype. Because no PTEN re-expression or downstream pathway-inhibition rescue experiment was performed, the PTEN–PI3K/AKT/mTOR axis should be considered a candidate signaling link rather than a demonstrated mediator of the observed phenotypes.

Because TET1 oxidizes 5-methylcytosine to 5-hydroxymethylcytosine, the reduction in PTEN after TET1 knockdown raises an epigenetic hypothesis (18, 19). PTEN promoter methylation has previously been reported in cervical neoplasia (20–22). However, PTEN promoter methylation, locus-specific 5-hydroxymethylcytosine enrichment, and TET1 occupancy were not examined in the present study. Direct epigenetic regulation of PTEN by TET1 therefore remains to be established. Several additional limitations should also be acknowledged. Functional experiments were performed in a single adenocarcinoma-derived cervical cancer cell line, and one shRNA with robust protein-level knockdown was used for downstream analyses. Rescue experiments, *in vivo* validation, Bobcat339 target-engagement measurements, and Bobcat339-treated downstream western blotting were not performed. In addition, the IHC cohort was small, and the public transcriptomic analyses were restricted to SCC whereas the tissue cohort comprised cervical adenocarcinomas. Although the latter is histologically consistent with the HeLa model, it limits direct comparison across the different study components. Bobcat339 should therefore be interpreted only as a complementary TET-family perturbation rather than TET1-specific validation. Future studies should incorporate additional SCC-derived cervical cancer models, mechanistic rescue experiments, locus-specific epigenetic assays, and larger independent tissue cohorts.

In conclusion, SCC-restricted public datasets support reduced TET1 expression in cervical SCC, while TET1 depletion promotes malignant phenotypes in HeLa cells. The accompanying reduction in PTEN expression and increase in PI3K/AKT/mTOR phosphorylation identify a candidate signaling association that warrants validation in additional models and mechanistic rescue studies.

## Data Availability

The original contributions presented in the study are included in the article/**Supplementary Material**. Further inquiries can be directed to the corresponding authors.
