# Supplementary material for: TET1 loss promotes malignant phenotypes in HeLa cells and is associated with altered PTEN–PI3K/AKT/mTOR signaling
Source: Front Oncol. 2026 Sep 24;16:1963427. doi: 10.3389/fonc.2026.1963427 (PMC13646133; doi:10.3389/fonc.2026.1963427)
Supplement: Supplementary file 1 [file SupplementaryFile1.docx]

**Supporting Information**

TET1 loss promotes malignant phenotypes in HeLa cells and is associated with altered PTEN–PI3K/AKT/mTOR signaling

Yan Liu^1^, Lujia Liu^1^, Lusheng Wang^2^, Juan Ren^1^, Fanle Meng^1^, Mengling Chen^1^, Xiaoyan Yao^1^, Ping Zou^3^, Jingjing Wu^3^, Xiaoyan Zhou^1^, Heng Tang^2, *^, Weiwei Ma^1, *^

^1^Obstetrics and Gynecology, Wanbei Coal Electric Group General Hospital, Suzhou, Anhui Province 234000, China, ^2^Central Laboratory, Wanbei Coal Electric Group General Hospital, Suzhou, Anhui Province 234000, China, ^3^Key Laboratory of Tumor Pathology of Suzhou, Suzhou, Anhui Province 234000, China

Figure **S1**. Patient-level pseudobulk associations in CNV-supported malignant epithelial cells. Spearman associations between patient-level TET1 expression and PI3K–AKT–mTOR, EMT, cell-cycle composite, and apoptosis scores in 12 cervical SCC patients. None survived Benjamini–Hochberg correction; analyses are exploratory.

Figure **S2**. Effect-size summary of patient-level pseudobulk associations. Points and intervals indicate Spearman ρ estimates and 10,000-resample bootstrap 95% CIs for associations between patient-level TET1 expression and PI3K–AKT–mTOR, EMT, cell-cycle composite, and apoptosis scores in 12 cervical SCC patients. FDR values were calculated by Benjamini–Hochberg correction across the four prespecified tests.

Figure **S3**. SCC-restricted TET1 expression in two public bulk cohorts. Points and intervals indicate Cliff’s δ estimates for SCC versus normal cervical tissue and bootstrap 95% CIs in GSE9750 (32 SCC versus 24 normal) and TCGA-CESC/GTEx (252 SCC versus 10 donor-level normal tissues).

Figure **S4**. TCGA-CESC SCC correlations between TET1 and prespecified transcriptomic pathway scores. Points and horizontal lines indicate Spearman ρ values and bootstrap 95% CIs; FDR values were calculated across the four pathway tests.

Figure **S5**. Association between TET1 and PTEN expression in TCGA-CESC cervical SCC. Spearman correlation was calculated across 252 SCC tumors (ρ = 0.081, 95% CI −0.055 to 0.211; P = 0.201).

Table **S1**. Clinicopathological characteristics and TET1 immunohistochemical H-scores of the paired cervical adenocarcinoma cohort

| Patient ID | Age (years) | Diagnosis | Adjacent non-neoplastic H-score | Tumor H-score |
| --- | --- | --- | --- | --- |
| P1 | 48 | Cervical adenocarcinoma with focal HSIL (CIN2) | 107.5 | 47.7 |
| P2 | 34 | Cervical adenocarcinoma | 107.0 | 29.2 |
| P3 | 56 | Usual-type cervical adenocarcinoma | 126.5 | 99.7 |
| P4 | 55 | Cervical adenocarcinoma | 124.7 | 24.4 |
| P5 | 60 | Cervical adenocarcinoma with focal HSIL (CIN2) | 209.0 | 6.7 |

Table **S2**. Quantitative cell-cycle distribution in HeLa cells

| **Group** | **G0/G1 (%)** | **S (%)** | **G2/M (%)** |
| --- | --- | --- | --- |
| sh-NC | 61.92 ± 1.59 | 25.61 ± 1.46 | 12.47 ± 0.27 |
| sh-TET1 | 54.61 ± 0.36 | 30.56 ± 0.77 | 14.84 ± 0.41 |
| sh-NC + Bobcat339 | 54.80 ± 0.82 | 31.13 ± 1.11 | 14.07 ± 0.37 |

Note: Data are presented as mean ± SD from three independent experiments. Each cell-cycle phase was analyzed separately using one-way ANOVA followed by Tukey’s multiple-comparisons test.
